# Supplementary material for: The impact of contact tracing and household bubbles on deconfinement strategies for COVID-19
Source: Nat Commun. 2021 Mar 9;12:1524. doi: 10.1038/s41467-021-21747-7 (PMC7943552; doi:10.1038/s41467-021-21747-7)
Supplement: Supplementary file 1 — Supplementary Information [file 41467_2021_21747_MOESM1_ESM.pdf]

The impact of contact tracing and household bubbles on deconfinement strategies for COVID-19 (SUPPLEMENTARY INFORMATION)

Lander Willem[1], Steven Abrams[2,3], Pieter J. K. Libin[2,4,5], Pietro Coletti[2], Elise Kuylen[1,2], Oana Petrof[2], Signe Møgelmose[2,6], James Wambua[2], Sereina A. Herzog[1,2], Christel Faes[2], Philippe Beutels[1,7], Niel Hens[1,2]

[1] Centre for Health Economic Research and Modelling Infectious Diseases, University of Antwerp, Antwerp, Belgium. [2] Data Science Institute, UHasselt, Hasselt, Belgium. [3] Global Health Institute, University of Antwerp, Antwerp, Belgium. [4] Artificial Intelligence Lab, Vrije Universiteit Brussel, Brussels, Belgium . [5] Rega Institute for Medical Research, Clinical and Epidemiological Virology, University of Leuven, Leuven, Belgium. [6] Centre for Population, Family and Health, University of Antwerp, Antwerp, Belgium. [7] School of Public Health and Community Medicine, The University of New South Wales, Sydney, Australia

Abstract

This document contains the supplementary information accompanying the manuscript "The impact of contact tracing and household bubbles on deconfinement strategies for COVID-19" by Willem et al.

Contents

S1 Model population . . . 2
S2 Social contact patterns . . . 4
S3 Age-specific probability to be symptomatic . . . 6
S4 Natural disease history . . . 7
S5 Transmission probability per contact . . . 10
S6 Hospital admission probability . . . 12
S7 Parameter estimation . . . 13
S8 Age-specific susceptibility . . . 16
S9 Scenario definitions . . . 19
S10 Robustness analyses . . . 20
S11 Ensemble analyses . . . 23
S12 Platform and technical details . . . 26
S13 References (Supplementary Information) . . . 26

S1 Model population

This work builds upon a stochastic individual-based simulator, STRIDE, we developed for influenza [1, 2] and measles [3]. Our individual-based model has a particular focus on social contact patterns by modelling each individual as part of "contact pools", representing a household, school-class, workplace, or community.

Household combinations, which specify the age of each member, are based on Belgian census data from 2011. We had to process the census data to ensure anonymization by excluding households containing 7 or more individuals (3.5% of population) and by using age groups for household combinations with a frequency of less than five. As such, we aggregated ages into 2-year intervals for individuals aged 0-25 and 5-year intervals for individuals over 26 years of age. If the frequency of an aggregated household composition still remained less than five, the households were excluded (0.7% of population). Next, we re-sampled ages from the age intervals to settle each household combination for our model population. We matched our resulting household data with summary statistics for household size and noticed an under-representation of large households containing children. Therefore, we duplicated 25,000 and 45,000 randomly chosen households of size 5 and 6, respectively, in which the second youngest household member was of age 0-25. These numbers were chosen to obtain matching distributions regarding household size, age in the population and age per household size. Supplementary Figures 1 and 2 present summary statistics from the model population and Belgian census data.

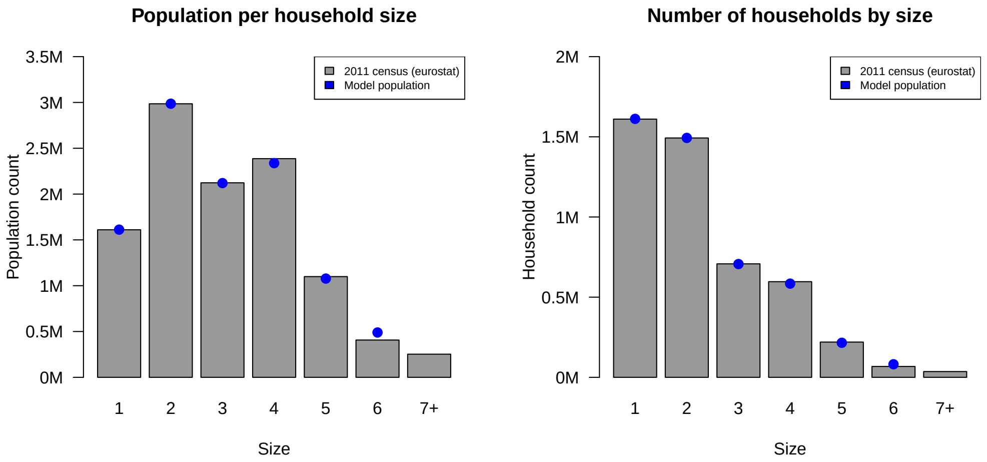

Supplementary Figure 1: Population count per household size and number of households per size: Belgian 2011 census and model population. Numbers are expressed in million (M).

We build our population by sampling households and assigning them to geographic locations based on the population census of 2001. Our population of 11 million people is closed, meaning that no births or deaths occur during the simulation, nor emigration or immigration. Children are assigned to a daycare center (0–2 years old), pre-school (3–5 years old), primary (6–11 years old), secondary (12–17 years old) or tertiary (18–23 years old) school based on Belgian enrolment statistics from Eurostat [4]. Daycare centers in Belgium comprise on average 8 infants [5], with a skewed distribution up to 18 infants. School classes in pre-, primary and secondary schools contain on average 19, 20 and 20 children, respectively, using a class size distribution based

on government statistics [6]. Students enrolled in tertiary schools are assigned to groups of 50 fellow students on average. Adults (18–64 years old) are assigned to a workplace or a daycare center/school class based on age-specific employment data and aggregated workplace size data from Eurostat [4]. We included one adult per 8 children in a daycare center and one adult per class in the pre- and primary school setting. Each “workplace” represents professional contacts in line with “business-to-business” (B2B) activities. The size of the workplaces is based on data from Eurostat and categorized into 1-9 (94%), 10-19 (3%), 20-49 (2%), 50-249 (0.8%) and +250 (0.2%) people. Geographic workplace assignment is based on commuting data from the Belgian 2001 census.

To represent leisure activities, family visits, “business-to-consumer” (B2C) and other contacts, the model contains “communities”. Each community is specified by a geographic center based on population density and contains on average 500 individuals. This arbitrary number affects contact probabilities but it does not influence contact rates. Each individual is assigned to one of three nearest community centers close to their home to represent weekday interactions and activities. For weekends, individuals can be assigned to the same community center or another one close to home. This community setup allows individuals to have similar contacts during week and weekend days, but prevents a strict compartmentalisation of the population.

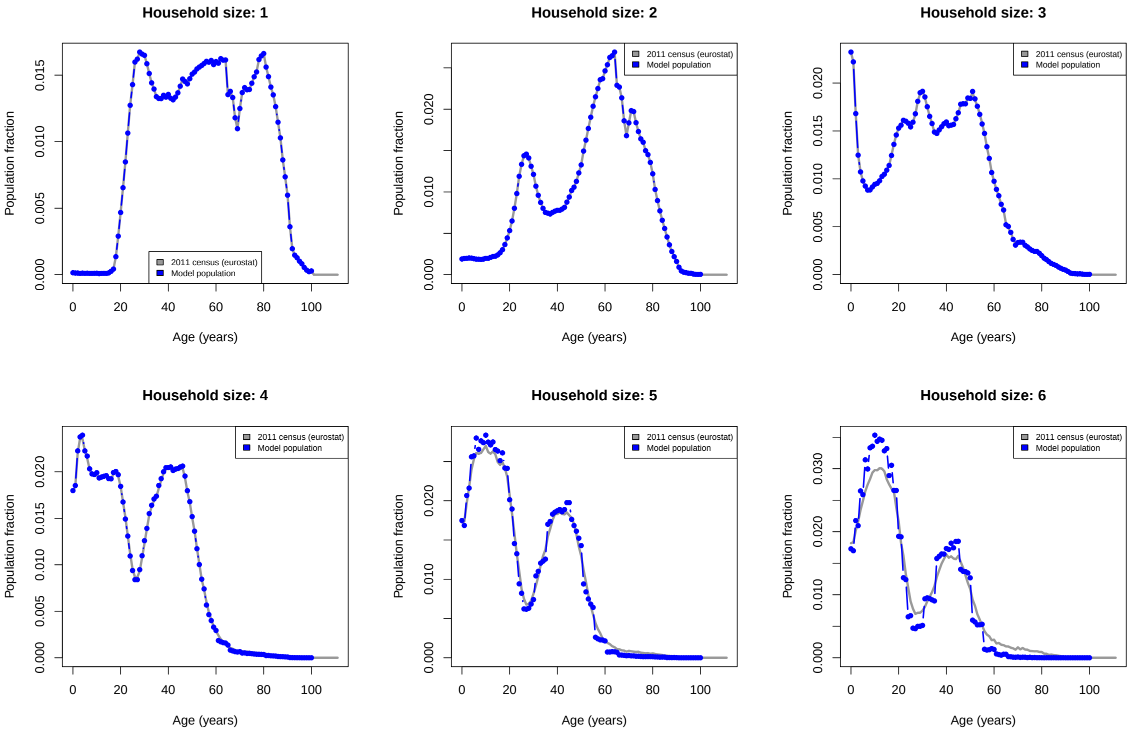

Supplementary Figure 2: Age profile per household size from the Belgian 2011 census and the model population.

S2 Social contact patterns

Social mixing in STRIDE is based on a diary-based social contact study performed in Belgium in 2010-2011 [7, 8, 9]. All participants were asked to record their contacts during one randomly assigned day. They also reported their time-use by activity, location and distance from home. Supplementary Table 1 provides an overview on how the survey data is used to inform contact pools in the individual-based model. Note that contact rates at school and at work are conditional upon school enrolment and employment, respectively. During each time step, we match the age- and location-specific contact rate (= number of contacts per day) with the number of individuals in a contact pool (e.g., workplace, school, etc.) to calculate the contact probability. We limit contact probabilities at 0.999, to prevent deterministic system behavior, and always use this maximum in a household setting since random mixing between household members is an adequate approximation of social contact behaviour for infections transmitted via close contacts [10]. Age-specific contact matrices are aggregated in the model into a vector containing age-dependent contact rates before calculating the contact probability. If people of different ages are part of the same contact pool, we use the lowest age-specific probability in their Bernoulli trial to enable a contact event.

The model allows to track social contacts in the population on a daily basis for all or for a random selection of individuals. The output is aligned with Socialmixr [11] and SOCRATES [12] to generate and visualise aggregated social contact matrices. Supplementary Figure 3 presents social contact patterns from STRIDE summarized in 2x2 matrices based on 5000 individuals during weekend days and Supplementary Figure 4 for weekdays before the COVID-19 lockdown.

Supplementary Table 1: Implementation of "contact pools" in STRIDE based on social contact survey data [7, 8, 9] Participants with more than 20 professional contacts per day (with students, clients, patients, etc.) had to report only the total number and age groups of their Supplementary Professional Contacts (SPC).

**Reported location in the social contact survey**	**Contact details in the social contact survey**	**Survey participant selection**	**Contact pool in STRIDE**
Home	Household members	All	Household
Home	Non-household members	All	Community
School	All	"Student" (or age <18y) and time-use data contains "school"	School
Work (B2B)	Adults (>17y), >15min and non-SPC	"Employed" and time-use data contains "work"	Workplace
Work (Teaching)	Children (0-11y)	"Employed" and time-use data contains "work"	School
Work (B2C and teaching)	non-B2B contacts	"Employed" and time-use data contains "work"	Community
Leisure, transport, family, grandparents, other	All	All	Community

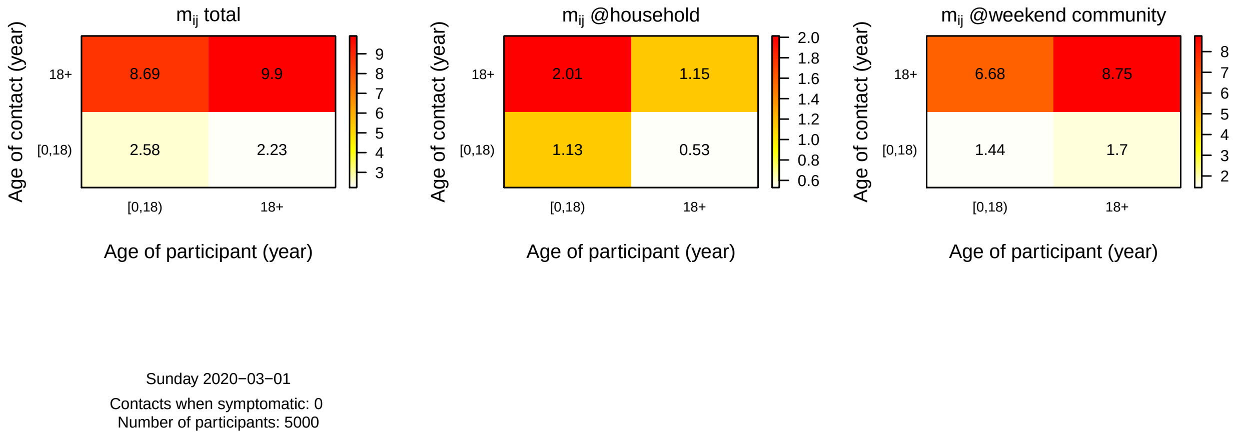

Supplementary Figure 3: Social contact matrices based on 5000 individuals within STRIDE during weekends. These are aggregated 2x2 matrices based on one-year age group data from the model.

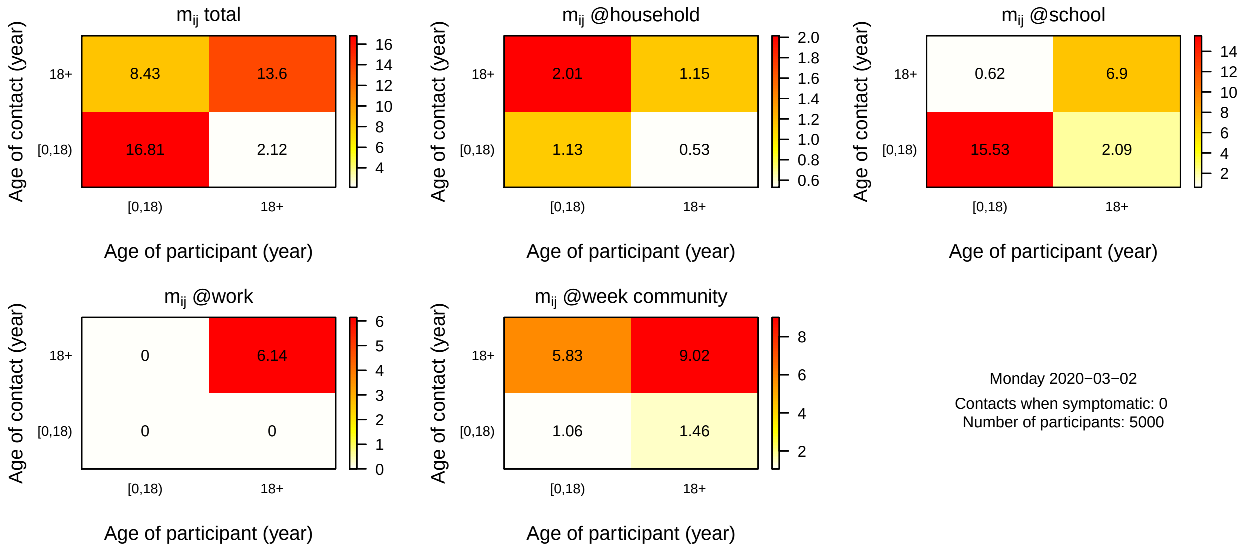

Supplementary Figure 4: Social contact matrices based on 5000 individuals within STRIDE during weekdays. These are aggregated 2x2 matrices based on one-year age group data from the model.

S3 Age-specific probability to be symptomatic

We assumed an overall proportion of symptomatic cases in the population before lockdown measures of 50% based on Li et al. [13]. To obtain an age-specific probability of symptomatic cases, we combined this population estimate with the age-specific relative susceptibility to symptomatic infection reported by Wu et al. [14]. Supplementary Figure 5 presents the age-specific data from [14], which we amended with an assumption for individuals 0-19 years of age. These relative proportions had to be re-scaled and weighted by age to end up with a population average of 50%. Therefore, we calculated the relative population size by age as:

$$\tilde{N_a} = N_a/N_{total} * T \tag{1}$$

with N_a the population size of age group a, N_{total} the total population size and T the number of age groups. Secondly, we calculated the age-specific probability to be symptomatic as:

$$P_a = \frac{S_a}{1/T * \sum_{k=1}^{T} S_k * \tilde{N_k}} * P_{population} \tag{2}$$

with S_a the relative susceptibility to symptomatic infection for age a, $\tilde{N_a}$ the relative population size for age a, and $P_{population}$ the proportion symptomatic cases on the population level. Supplementary Figure 5 presents the resulting probabilities to be symptomatic by age. Note that we had to truncate the highest relative susceptibility to symptomatic infection to maintain all age-specific probabilities between 0 and 1. This limitation is due to the interaction between the age-specific susceptibility and population sizes with the overall proportion of 50%.

The proportion of symptomatic cases per age group is disease-related and fixed over time. The proportion of symptomatic cases in the population depends on the age of the newly infected cases, which is driven by social contact and transmission dynamics. Given the temporal aspects of social contact behavior and restrictions, the overall proportion of symptomatic cases in the population can change over time.

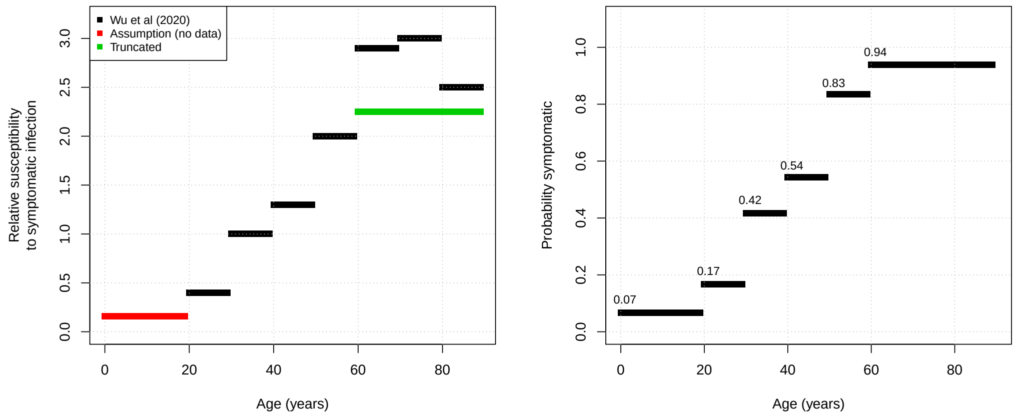

Supplementary Figure 5: Relative susceptibility to symptomatic infection by age based on Wu et al. [14] (left) and estimated probability of symptomatic infections (right).

S4 Natural disease history

The individual-based model STRIDE for COVID-19 is based on susceptible, exposed, infectious and recovered individuals, with the infectious health state divided in pre-symptomatic, symptomatic and asymptomatic. Throughout this text, we use “asymptomatic” for all cases that do not experience any symptoms throughout their infection. We assume that the latent and infectious period for asymptomatic cases is similar to symptomatic cases, so the estimations for the start and duration of the infectious period are generalized for asymptomatic cases. The level of infectiousness increases after symptom onset. Supplementary Figure 6 presents the overall disease dynamics. Hospitalization is not part of the transmission model and added with a dashed line in combination with the age-specific rate based on a proportionality and delay factor.

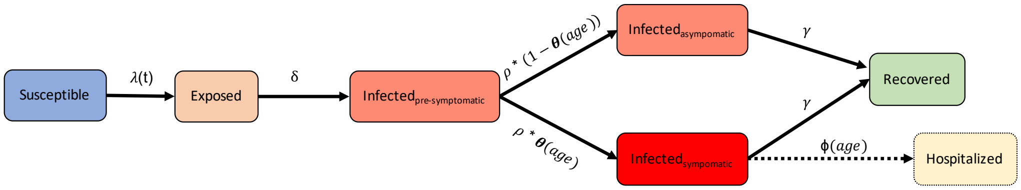

Supplementary Figure 6: Overall disease dynamics The infection process is driven by the force of infection $\lambda(t)$, latent period $1/\delta$, pre-symptomatic period $1/\rho$, proportion symptomatic $\theta(age)$, recovery rate γ and hospitalization rate $\phi(age)$.

From the individual perspective (see Supplementary Figure 7), each infected case experiences an incubation period, an infectious period and optionally a symptomatic period. The start of the infectious period is related to the incubation period and this dependency is captured in our model with a “pre-symptomatic” infectious period rather than characterising the latent period. This modelling choice is driven by the literature on the incubation period and pre-symptomatic infectiousness [15, 13]. Hospital admissions are calculated post-hoc based on the model output. For each symptomatic case, there is a likelihood to be hospitalised and a delay distribution to specify the time between symptom onset and hospital admission. This likelihood and delay are age-specific and are described in Section S6.

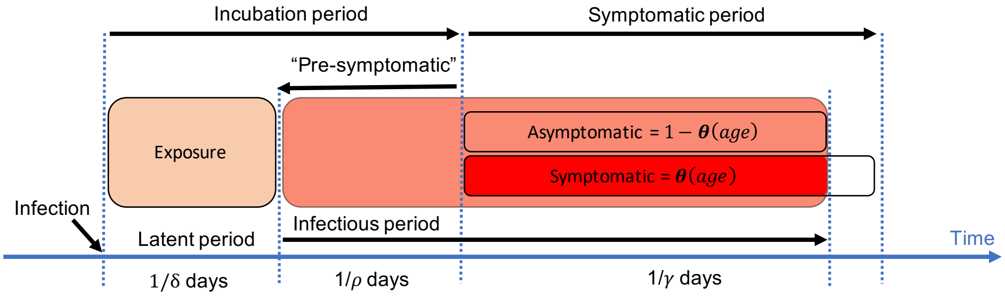

Supplementary Figure 7: Disease dynamics in the STRIDE model on the level of the individual. Exposed individuals become infectious without symptoms after $1/\delta$ days. Since not all cases develop symptoms $1/\rho$ days later, we denote this second stage as “pre-symptomatic”. After $1/\gamma$ days, individuals are not infectious anymore. Disease parameters are described in Supplementary Table 2.

In practice, we define for each infected case an incubation period and whether the case is symptomatic or asymptomatic. Relative to the end of the incubation period, we specify the start of the infectious period and the duration. The following paragraphs describe how we derive the probability functions for the incubation, pre-symptomatic, infectious and symptomatic period.

The start of the infectious period, relative to symptom onset, is based on the results from He et al [15], who concluded that viral shedding may begin 5 to 6 days before the appearance of the first symptoms. They conclude that after symptom onset, viral loads decrease monotonically and decline significantly 8 days after symptom onset, as live virus could no longer be cultured. The inferred infectiousness profile was captured in a shifted gamma distribution with shape 20.52, rate 1.59 and shift 12.27 days. To estimate the pre-symptomatic infectious period density for STRIDE. We truncated the shifted gamma distribution at -1 by dividing the probability density function by the cumulative distribution function evaluated at -1 (left panel of Supplementary Figure 8).

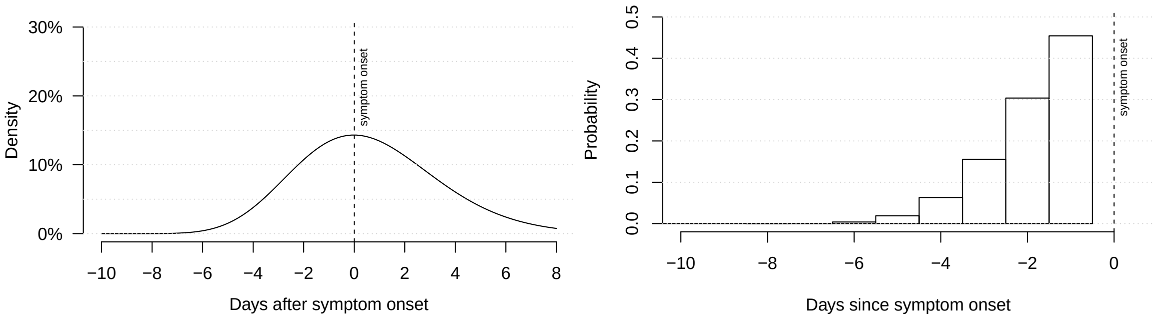

Supplementary Figure 8: Infectiousness profile relative to symptom onset from He et al [15] (left) and the discrete pre-symptomatic infectious period distribution for STRIDE (right).

The incubation period in STRIDE is based on the reported mean of 5.2 days (95% confidence interval: 4.1 to 7.0) from Li et al. [13]. The distribution follows a log-normal distribution with logmean=1.43 and logsd=0.66. The numerical parameters are derived from He et al. [15] and are represented by the density plot in Supplementary Figure 9. We calculated the discrete version of this log-normal distribution with the assumption that infectiousness has to start at least one day prior to symptom onset and at least one day after infection. As a result, the incubation period is at least 2 days, which results in the probability distribution as presented in Supplementary Figure 9;

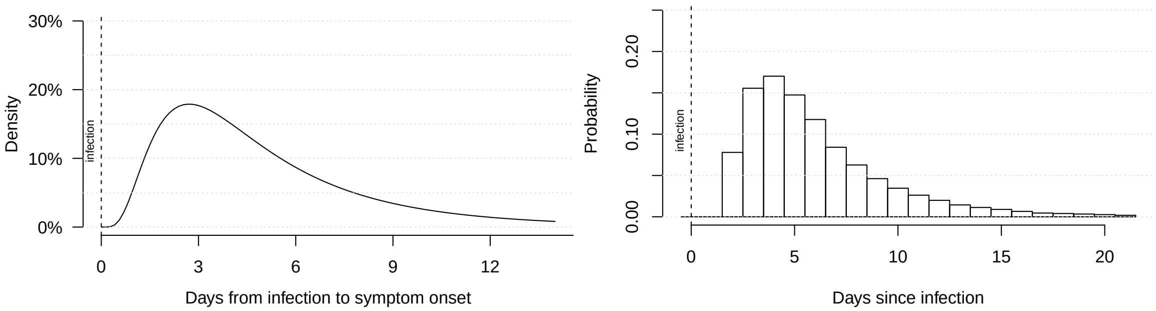

Supplementary Figure 9: Incubation period distribution from Li et al. [13] (left) and the standardized discrete distribution for STRIDE (right).

He et al. [15] reports that infectiousness declines after symptom onset up to a maximum of 7 days, which restricts the overall infectious period. The modelling study by Lourenco et al. [16] used a normal distribution with a mean of 4.5 days and standard deviation of 1. Given their SIR model structure, this value cannot be used directly, but was used as a prior for our parameter estimation. We evaluated normal distributions with mean 5, 6 and 7 days and standard deviation 1 with the aim that the combination of the pre-symptomatic period and the total infectious period resembles the infectious profile relative to symptom onset from He et al.[15]. With a mean infectious period of 6 days, the number of individuals in STRIDE that are still infectious 7 and 8 days after symptom onset corresponds to 3% and <1%, respectively, which is in line with the results [15]. The infectious period distributions relative to infection and symptom onset are presented in Supplementary Figure 10.

The focus of the transmission model lies on new infections over time and the behaviour of symptomatic cases after their infectious period has no impact on transmission dynamics. Therefore, we choose to fix the symptomatic period to the maximum infectious period after symptom onset to 7 days. The infectiousness of asymptomatic cases in STRIDE is set to 50% compared to symptomatic cases, based on Li et al. [13].

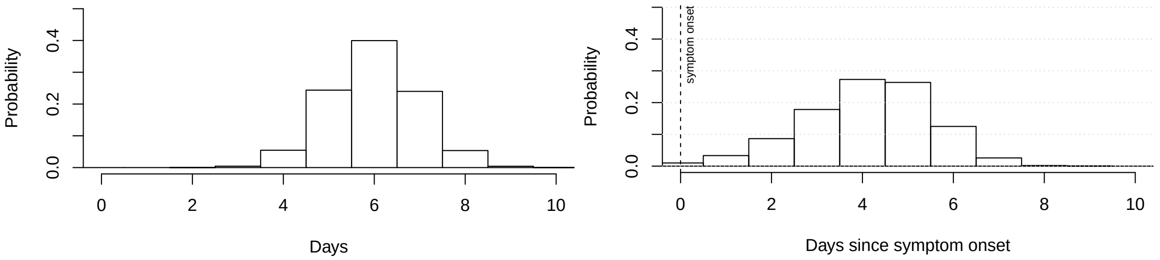

Supplementary Figure 10: Infectious period distribution in STRIDE in total (left) and relative to symptom onset (right)

Supplementary Table 2: Summary table with disease characteristics. Transition parameters are discretized to the modelling time step of 1 day

Parameter	Model value	Reference
Incubation period	Lognormal(logmean=1.43, logsd=0.66)	[15]
Symptomatic period	7 days, which is the maximum infectious period after symptom onset	[15]
Infectious period	Normal(mean=6, sd=1)	[15, 16]
Pre-symptomatic infectious period	Gamma(shape=20.52, rate=1.59, shift= 12.27) truncated at -1 and divided by the cumulative distribution function for standardisation	[15]
Probability to be symptomatic (age-specific)	Section S3	[17, 14]
Infectiousness asymptomatic cases	50% of symptomatic cases	[13]

S5 Transmission probability per contact

The basic reproduction number (R_0) captures the combined effect of social contact patterns, demography, disease dynamics and the probability of transmission given contact. By keeping everything constant except the transmission probability, we can fit the relation with R_0 by counting the number of secondary cases per index case in a susceptible population. The goal is to easily translate a transmission probability given contact to R_0 and vice versa. Especially the latter is convenient to specify R_0 as model parameter. As such, a given R_0 is converted into a transmission parameter to be used in the simulation model. To enable this conversion, we need a continuous function for the number of secondary cases in terms of the transmission probability, which we can invert.

For the entire range of transmission probabilities, this relation needs to go through zero and levels off by the total number of unique contacts within the infectious period for the simulated population, social contact dynamics and disease characteristics. Capturing the full relation between the transmission probability and the secondary cases falls outside the scope of this research, since our goal is to simulate slight variations of the transmission probability to obtain R_0 values in range with published values. As such, we focus on a specific range of the transmission probability and assume a linear relation between the transmission probability and the average number of secondary cases in a susceptible population. Based on preliminary runs, we defined a range for the transmission probability between 0.01 and 0.14, which corresponds with an average number of secondary cases between 1 and 5.

Each simulation with a specified transmission probability started with 20 randomly infected cases between 1 and 99 years of age, and traced their secondary cases. Note that newborns are never selected as index cases. The choice for 20 infected cases instead of 1 in a fully susceptible population according the definition of R_0 is to reduce the number of model realisations with factor 20. On 11 million people, the difference between 1 and 20 infected seeds is inferior. To capture temporary effects for (pre-)symptomatic infectious periods during week and weekend days, we ran different simulations for each transmission probability starting from Monday the 1st up to Sunday the 7th of February 2020.

For each transmission probability (15x) and starting day (7x), we ran 10 stochastic realisations starting with 20 infected cases. As such, our fitting procedure to capture the relation between the transmission probability and the (basic) reproduction number is based on the secondary cases of 21000 index cases. Supplementary Figure 11 presents the results of these simulations and the linear model we fitted through these data:

$$\text{Secondary cases in a susceptible population} = R_0 = 39.65 * P_{transmission} + 0.12$$

To use R_0 as model input, we use the inverse:

$$P_{transmission} = R_0/39.65 - 0.12$$

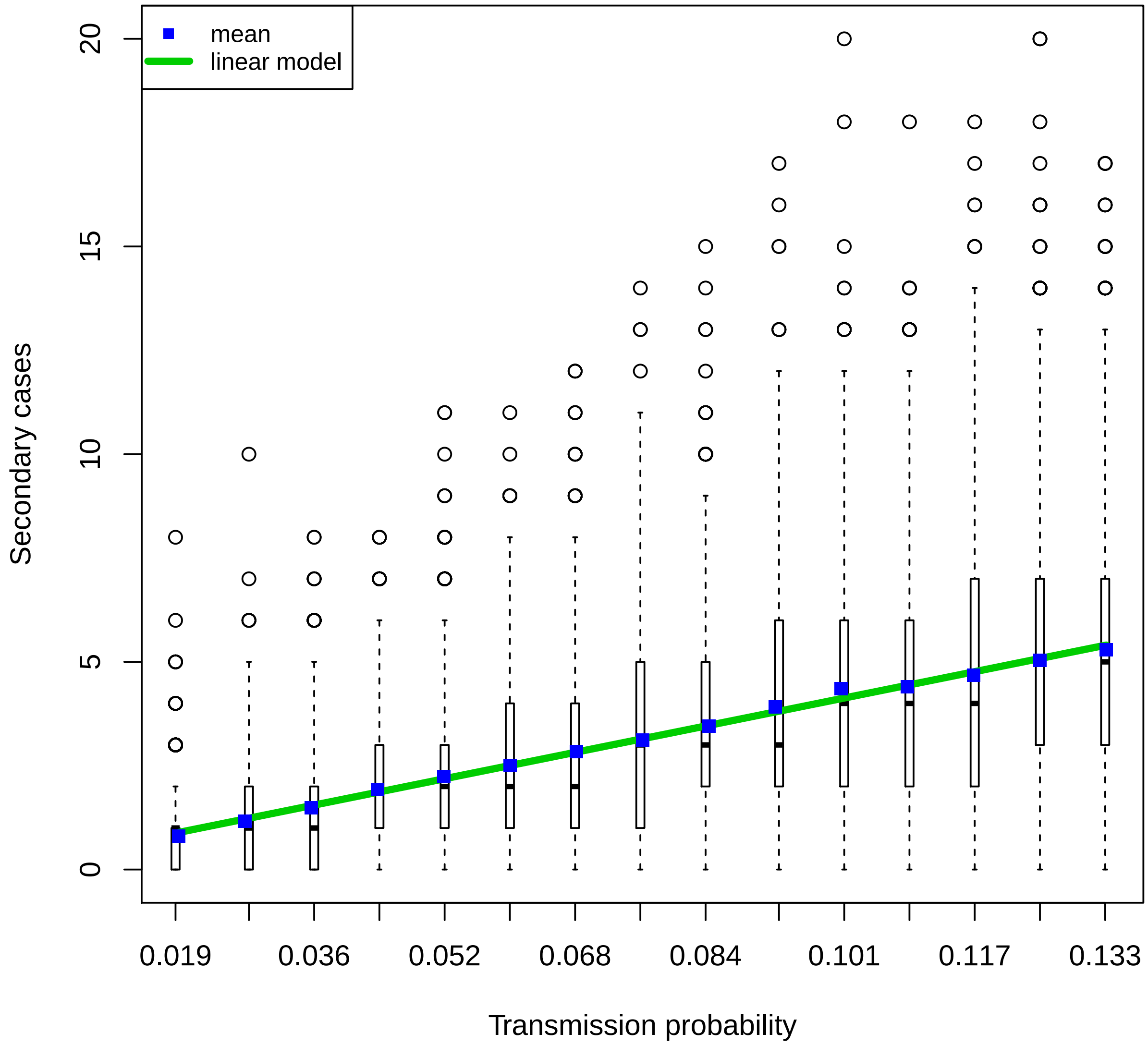

Supplementary Figure 11: The distribution of secondary cases of index cases in a susceptible population by transmission probability. Each boxplot presents the number of secondary cases of 1400 index cases by the median, quartiles (box), 2.5 and 97.5 percentiles (whiskers) and outliers (circles).

S6 Hospital admission probability

To estimate time-invariant relative hospital admission probabilities by age, we used the reported hospital admissions up to week 13 (March 30th, 2020) from a Belgian hospital survey [18]. We assumed that hospitals admissions up to week 13 are little affected by the lockdown at the end of week 11 given the mean incubation period of 5.2 days [13], the mean time between symptom onset and hospital admission of 5.6 days [18]. Supplementary Table 3 present the relative proportion of hospitalized cases by age, averaged for week 11-13. This selection of hospital admission data enables to use a denominator for the number of cases that is not affected by the lockdown, which is the topic of interest in this study. We used the age-specific proportion of symptomatic cases from the simulations to estimate the relationship between the transmission probability and the secondary cases. As such, the calculated relative hospital probabilities are independent from R_0 and COVID-19 related interventions. We stratified the number of symptomatic cases by 10-year age categories and present them in Supplementary Table 3. We calculated the relative fraction of symptomatic cases to be hospitalized and a standardized version relative to the oldest age group. These relative proportions are subject to a scaling factor, which we estimated during the parameter estimation procedure as reported in section S7.

Supplementary Table 3: Age-specific proportionality of hospital admission in Belgium based on averaged hospital survey data from week 11-13 and simulated symptomatic cases infected before physical distancing measures occurred. The hospital delay represents the time between symptom onset and hospitalization [18].

Age category	Reported hospital admissions	Simulated proportion symptomatic cases	Relative fraction	Relative fraction (standardized)	Hospital delay
0-9y	1.4%	2.0%	0.72	0.091	3 days
10-19y	0.2%	3.2%	0.07	0.009	3 days
20-29y	1.8%	5.2%	0.35	0.044	7 days
30-39y	3.9%	14.7%	0.26	0.033	7 days
40-49y	9.6%	21.5%	0.45	0.057	7 days
50-59	15.2%	25.9%	0.59	0.075	7 days
60-69y	18.5%	16.4%	1.13	0.143	6 days
70-79y	22.7%	7.7%	2.93	0.373	6 days
+80y	26.7%	3.4%	7.87	1.000	1 day

S7 Parameter estimation

We estimated transmission and lockdown characteristics based on reported hospital admissions, initial doubling time and serial sero-prevalence data up to May 1st. Afterwards, multiple restrictive measures in Belgium were relaxed, which is the focus of our scenario analysis. From March 14th onward, people who could, were coerced into telework and many businesses, unable to guarantee required hygiene and physical distancing measures had to close. All business-to-consumer (B2C) outlets were interrupted except for ordered home deliveries and shops selling essential goods. All schools were closed except for a low number of children that required day care (e.g., if both parents were not able to telework), so we assumed neither social contacts nor transmission at schools.

We estimated the reduction in social mixing associated with business-to-business (B2B) activities, and in the community (including B2C). An instant reduction from March 14th did not provide a good fit with observed data. Therefore, we included a linear increase in compliance to reduce social contacts. The level of reduction and the duration for this compliance to reach the max is the subject of our parameter estimation.

Disease and lockdown parameters:

- Transmission probability per contact, which we express as R_0 using the linear model from Section S5.
- The number of introductions into the population and the timing. Since these two parameters are strongly correlated, we choose to fix the introduction date to Monday February 17th, 2020, which is one month prior the study period (lockdown) to allow the model to have some burn-in period without starting within the spring break (February 24-28, 2020).
- Hospital probability scaling factor, to use in combination with the age-specific relative hospital probability values as presented in Supplementary Table 3.
- Social contact reductions at workplaces and the delay to reach full compliance after March 14th, 2020. We assumed that the compliance increased linearly over time.
- Social contact reductions in the community and the delay to reach full compliance after March 14th, 2020. We assumed that the compliance increased linearly over time.

Reference data

- Total hospital admissions per day as reported by the Belgian Health Institute Sciensano [19].
- Doubling time pre-lockdown of 3.1 (2.4-4.4), based on [20]. From each simulation we used the average doubling time between February 24th up to March 8th, 2020.
- Serial sero-prevalence of 0.029 (0.023-0.036) on March 19th and 0.060 (0.051-0.071) on April 9th, 2020, based on [21]. These seroprevalence rates are derived from samples collected during one week starting on March 30th and April 20th. We used the midpoint of these sample weeks and assume that the infections took place at least 14 days earlier in order to reflect the minimum time needed to build up IgG antibodies against SARS-CoV-2 that can be detected by ELISA tests [21].

We used the Poisson log-likelihood statistic to assess how well the model describes the reference data. This method is appropriate when dealing with count data, such as the hospital admissions [22]. The log-likelihood when ignoring a constant $log(k_i!)$ is calculated as

$$-logL = \sum_{i=1}^{N}(-k_i * log(\lambda_i) + \lambda_i)$$

with k_i the observed incidence and λ_i the predicted incidence on time point i over the time horizon N.

Multi-criteria and iterative procedure. Given our interest in hospital admissions, incidence and transmission dynamics, we used three reference outcomes or criteria for which we were able to calculate the log-likelihood. To select optimal parameter combinations, we used the intersection of the 15% best scoring model runs for each criteria. If this intersection contained less than 10 parameter sets, the cutoff was incremented by 2% until at least 10 parameter sets were included in our model ensemble. We started from broad parameter ranges to performed the multi-criteria procedure and used the resulting parameter ensemble to guide the parameter ranges for the subsequent iteration. This process was repeated up to 3 iterations using 1000 Latin Hypercube samples with 5 stochastic realisations. The initial and selected parameter ranges for each iteration are given in Supplementary Table 4.

After the first iteration, only the parameter range of R_0 substantially changed, which expresses the main role of this parameter given the initial ranges. During subsequent iterations, the importance of other parameters increased so they could weigh on the ensemble selection. The limited impact of the 3rd iteration made clear we reached a plateau in the parameter estimation procedure. We increased the number of stochastic realisations to 10 to select a final model parameter ensemble. From the latter, which takes all criteria into account, we selected the on average best scoring parameter set according the hospital admission data since this is the model outcome under study for the scenario analyses.

Simulations with more than 1500k cases up to May 1st were stopped and as such excluded from post-processing because this is twice the reported serial sero-prevalence in April 2020.

Supplementary Table 4: Parameter ranges and observed initial doubling time (min-max) along the estimation procedure and the final model parameter set. The hospital probability factor refers to the +80-age group. For the other age groups, see table 3.

**Parameter**	**Start**	**Iteration 1**	**Iteration 2**	**Iteration 3**	**Iteration 4**	**Final set**
R_0*	1-5	3.14 - 3.79	3.35 - 3.51	3.41 - 3.50	3.41 - 3.49	3.42
Infected introductions	200 - 600	121 - 556	225 - 321	233 - 308	236 - 307	263
Hospital probability factor	0.05 - 0.9	0.20 - 0.81	0.26 - 0.31	0.35 - 0.47	0.35 - 0.46	0.4
Contact reduction: workplace	0.60 - 0.95	0.62 - 0.95	0.65 - 0.94	0.67 - 0.94	0.70 - 0.93	0.86
Compliance delay: workplace (days)	5, 6, 7	5, 6, 7	5, 6, 7	5, 6, 7	5, 6, 7	7
Contact reduction: other	0.60 - 0.95	0.68 - 0.95	0.78 - 0.89	0.81 - 0.88	0.82 - 0.87	0.85
Compliance delay: other (days)	5, 6, 7	5, 6, 7	5, 6, 7	5, 6, 7	5, 6, 7	7
Latin Hypercube samples	-	1000	1000	1000	500	-
Stochastic realisations	-	5	5	5	10	-
Doubling time** (days)	-	2.81 - 3.43	3.04 - 3.21	3.01 - 3.16	3.00 - 3.18	3.09 - 3.17

* R_0 is used as input parameter by the reverse calculation to the transmission probability per contact; ** model output.

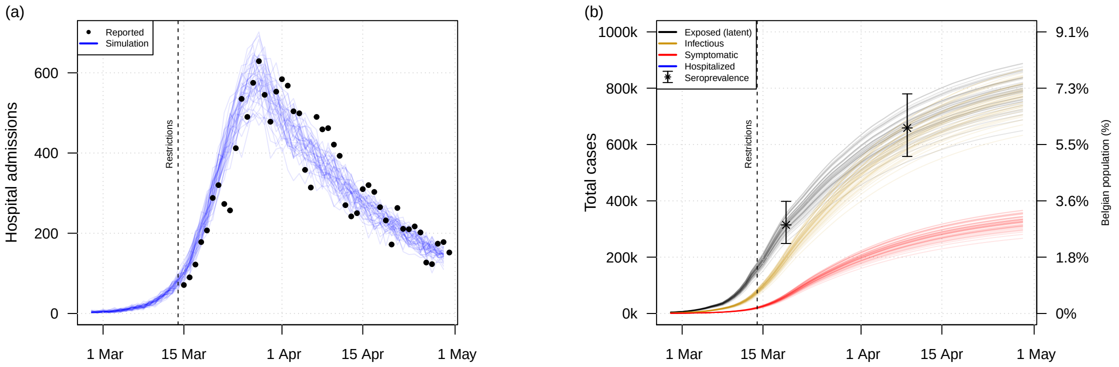

Supplementary Figure 12: Stochastic simulation results in terms of daily hospital admissions (a) and cumulative incidence of exposed, infectious and symptomatic cases over time (b) using the parameters from the "Final set" in Supplementary Table 4. The cumulative incidences are presented together with prediction intervals for March 19th and April 9th based on serial seroprevalence data (n=7307) as described in [21].

S8 Age-specific susceptibility

In our default model configuration, the infectiousness of children in the population is limited due to their low probability to be symptomatic and our assumption that asymptomatic cases are only 50% as infectious [13]. Hence, the probability to be symptomatic for children aged 0-19y is only 7%. To incorporate additional age-specific effects, we re-calibrated our transmission model given that children (0-17y) are only half as susceptible compared to adults (+18y). This change in disease characteristics required an update of the relationship between the transmission probability per contact and the number of secondary cases (i.e. the reproduction number given age-specific susceptibility, R_0^c). The procedure as described in Section S5 has been applied with the altered disease characteristics and resulted into:

$$R_0^c = 34.57 * P_{transmission} + 0.07$$

and the inverse:

$$P_{transmission} = R_0^c/34.57 - 0.07$$

We also re-estimated the relative hospital probability by age since the proportion of symptomatic cases before the lockdown changed. The results are presented in Supplementary Table 5. Finally, we repeated the iterative parameter estimation procedure as described in Section S7, and present the results in Supplementary Table 6. The resulting model fit is presented in Supplementary Figure 13.

Simulations for the baseline scenario with age-specific susceptibility in terms of hospital admissions and reproduction number (Supplementary Figure 14) are similar to the results presented in the main text (Supplementary Figure **??**). Also the simulations with household bubbles, contact tracing and a combined strategy scenario analyses are not much affected by the susceptibility assumption (Supplementary Figure 15). For strategies involving school re-opening, the effect is substantial as discussed in the main text.

Supplementary Table 5: Age-specific proportionally of hospital admission when children (0-17y) are only 50% as susceptible compared to adults (+18y). The hospital delay represents the time between symptom onset and hospitalization [18].

Age category	Reported hospital admissions	Simulated symptomatic cases	Fraction	Fraction (standardized)	Hospital delay
0-9y	1.4%	1%	1.71	0.127	3 days
10-19y	0.2%	2%	0.12	0.009	3 days
20-29y	1.8%	6%	0.33	0.024	7 days
30-39y	3.9%	15%	0.26	0.019	7 days
40-49y	9.6%	22%	0.44	0.033	7 days
50-59	15.2%	27%	0.56	0.041	7 days
60-69y	18.5%	17%	1.10	0.081	6 days
70-79y	17.0%	7.7%	2.20	0.163	6 days
+80y	45.8%	3.4%	13.51	1.000	1 day

Supplementary Table 6: Parameter ranges and observed initial doubling time (min-max) along the estimation procedure with age-specific susceptibility and final model parameter set. The hospital probability factor refers to the +80-age group. For the other age groups, see table 5.

Parameter	Start	Iteration 1	Iteration 2	Iteration 3	Iteration 4	Final set
R_0*	1-5	3.00 - 3.48	3.35 - 3.45	3.36 - 3.43	3.37 - 3.45	3.37
Infected introductions	200-600	206 - 593	210 - 322	230 - 389	217 - 279	225
Hospital probability factor	0.05 - 0.9	0.14 - 0.75	0.24 - 0.44	0.36 - 0.47	0.31 - 0.38	0.35
Contact reduction: workplace	0.60 - 0.95	0.61 - 0.94	0.64 - 0.90	0.69 - 0.92	0.68 - 0.89	0.76
Compliance delay: workplace (days)	5, 6, 7	5, 6, 7	5, 6, 7	5, 6, 7	5, 6, 7	6
Contact reduction: other	0.60 - 0.95	0.70 - 0.94	0.83 - 0.89	0.83 - 0.92	0.83 - 0.88	0.86
Compliance delay: other (days-	5, 6, 7	5, 6, 7	5, 6, 7	5, 6, 7	5, 6, 7	7
Latin Hypercube samples	-	1000	1000	1000	500	
Stochastic realisations per set	-	5	5	5	10	
Doubling time** (days)	-	3.03 - 3.54	3.04 - 3.19	3.09 - 3.11	3.00 - 3.20	3.09 - 3.19

* R_0 is used as input parameter by the reverse calculation to a transmission probability per contact; ** model output.

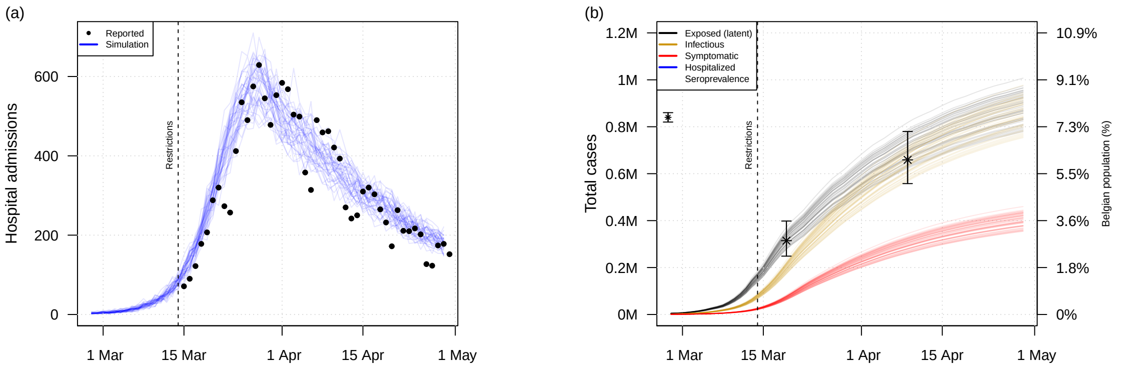

Supplementary Figure 13: Stochastic simulation results with age-specific susceptibility in terms of daily hospital admissions (a) and cumulative incidence of exposed, infectious and symptomatic cases over time (b) based on the parameters from the "Final set" in Supplementary Table 6. The cumulative incidences are presented together with prediction intervals for March 19th and April 9th based on serial seroprevalence data (n=7307) as described in [21].

R

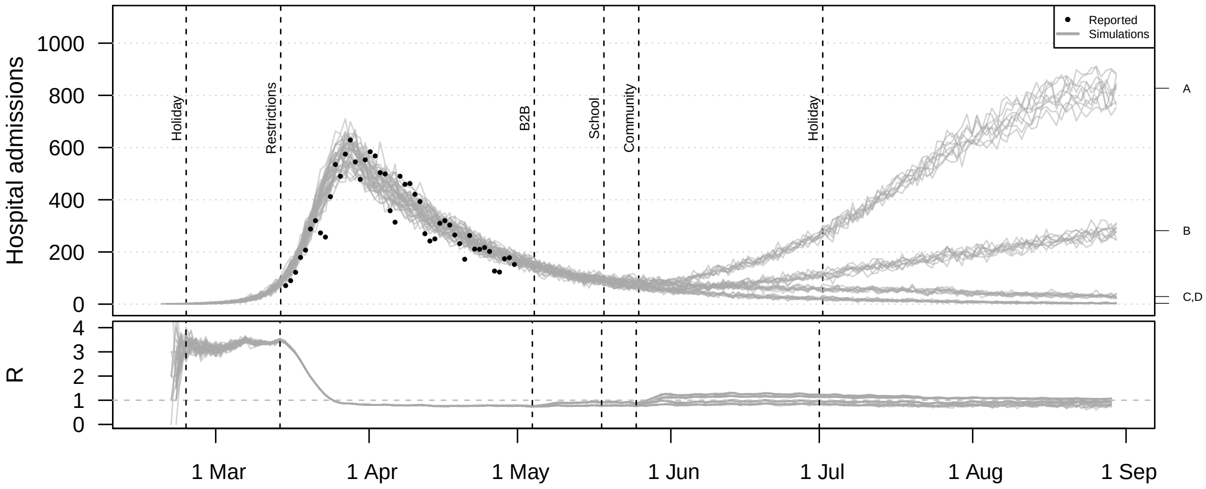

Supplementary Figure 14: Hospital admissions and effective reproduction number (R) from the baseline scenario including 4 mixing assumptions with age-specific susceptibility. All simulations include social restrictions from March 14th and the partial school reopening in May. For the B2B, the social mixing after the lockdown is assumed to double from the indicated point in time (indicated with A and C) or to remain constant (B,D). Social mixing in the community is assumed to double (A,B) or to remain constant (C,D).

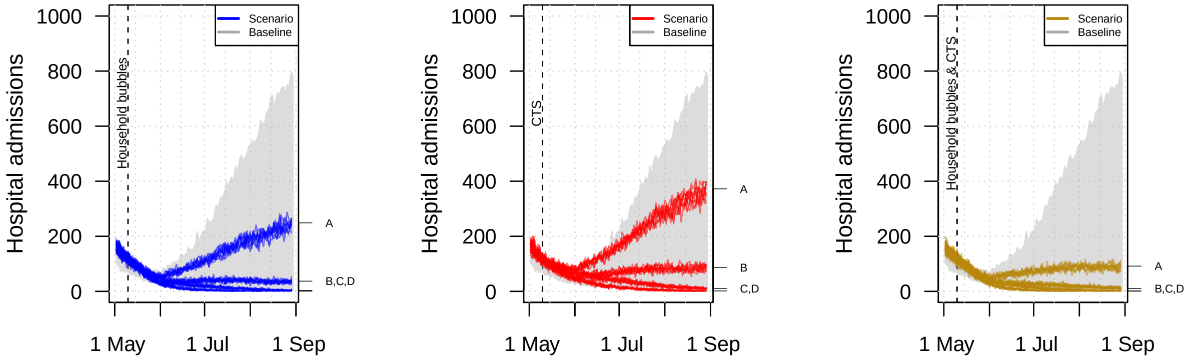

Supplementary Figure 15: Hospital admissions over time when community mixing occurs in household bubbles (left), a contact tracing strategy (CTS) is in place (center), or both (right) with age-specific susceptibility. All scenarios are based on the same natural disease history and quantitative mixing assumptions but differ from the baseline in terms of the network structure and application of contact tracing from the given point in time. The mixing assumptions A,B,C,D are explained in the caption of Supplementary Figure 14.

S9 Scenario definitions

We defined deconfinement scenarios by combinations of location-specific social mixing relative to pre-pandemic observations. By varying percentages of contacts at different locations, we implicitly assumed people either make fewer contacts compared to the pre-pandemic situation or the contacts they made were less likely to lead to transmission. For example, if we increased social mixing at workplaces from 25% to 50%, we predict the impact of "what if the risk to acquire infection at work doubled compared to during lockdown, though still 50% less than in pre-pandemic times". To account for structural uncertainty with respect to B2B-related social mixing, we included 25% and 50% of the social contacts rates prior lockdown. For community-related mixing, we used 15% and 30% of the social contact rates prior lockdown. By combining these B2B and community mixing patterns, we ended up with four social mixing parameter sets and we ran 10 stochastic realisations per parameter set. Supplementary Table 7 contains temporal aspects of the scenarios and the age-specific "school*" program based on the Belgian regulations.

Supplementary Table 7: Overview of lockdown measures and gradual relief in the scenario analyses.

**What?**	**Timing?**
Start restriction measures by closing schools, universities, cultural activities, bars and restaurants. Four days later, additional measures were taken to allow only work-related transport of essential workers, and teleworking made the norm.	March 14th
Re-start business-to-business (B2B)	May 4th
Social mixing in household bubbles	May 11th
Start contact tracing strategies (CTS)	May 11th
Re-open school* for 0-2year (daycare) & 6-7year (1st and 2nd grade primary school)	May 18th, 4 days / week
Re-open school* for 11year olds (6th grade primary school)	May 18th, 2 days / week
Re-open school* for 17year olds (6th grade secondary school)	May 18th, 1 day / week
Re-open schools for 0-5year, 0-11year, 0-17year	May 18th, 5 days / week
Increasing leisure, B2C and other social contacts (community)	May 25th

* Age-specific school-reopening as stated by the Belgian government on April 24th.

S10 Robustness analyses

There is no golden standard for the number of stochastic realisations for this type of stochastic simulator. The results in the main text are based on 10 realisations, but we present here a robustness analysis for the main scenarios (baseline, household bubbles, CTS and combined strategy) based on 20, 40 and 80 realisations. We did observe stochastic changes for the projected hospital admissions over time (Supplementary Figure 16 and 17) but no differences on the averages and average differences in terms of total hospital admissions (Supplementary Figure 18) with an increasing number of stochastic realisations.

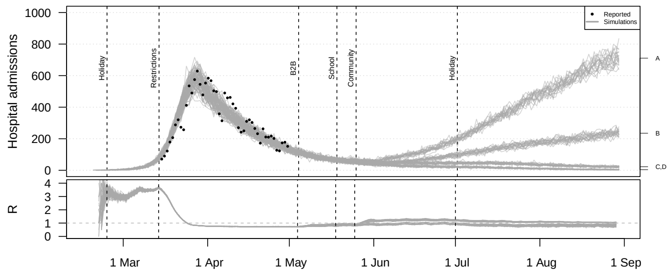

(a) Simulation results based on 20 stochastic realisations.

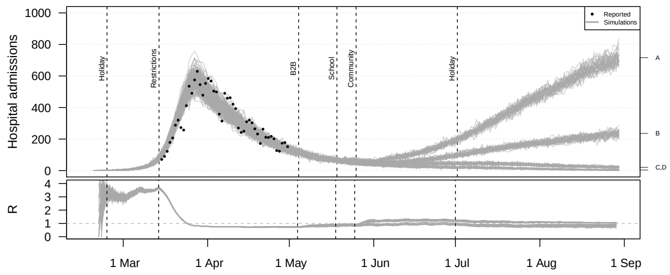

(b) Simulation results based on 40 stochastic realisations

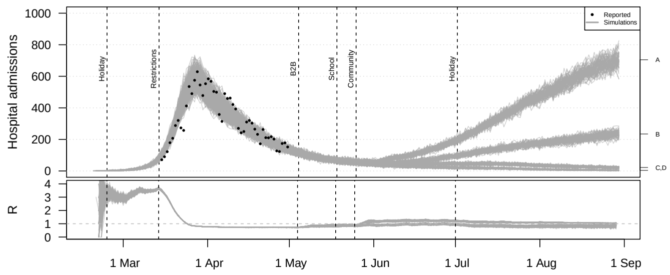

(c) Simulation results based on 80 stochastic realisations

Supplementary Figure 16: Hospital admissions over time for the baseline scenario based on 20, 40 and 80 stochastic realisations per social contact assumption. All simulations include social restrictions from March 14th and the partial school reopening in May. For the B2B, the social mixing after the lockdown is assumed to double from the indicated point in time (indicated with A and C) or to remain constant (B,D). Social mixing in the community is assumed to double (A,B) or to remain constant (C,D). The dots present the reported hospital admissions for Belgium.

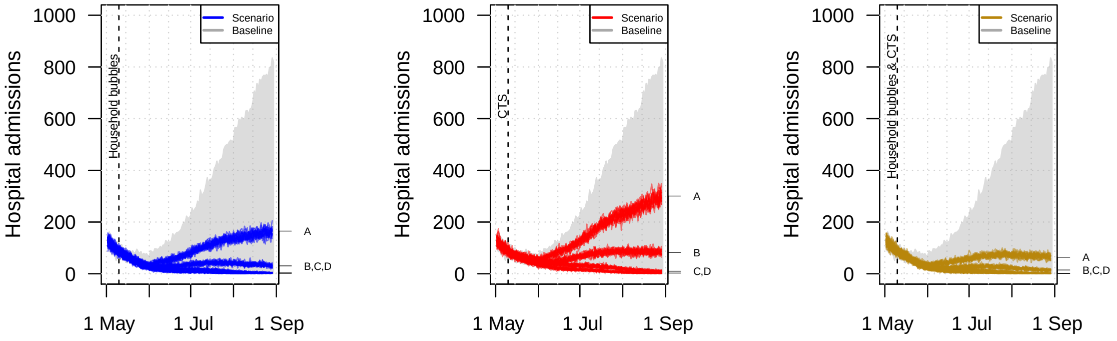

(a) Simulation results based on 20 stochastic realisations.

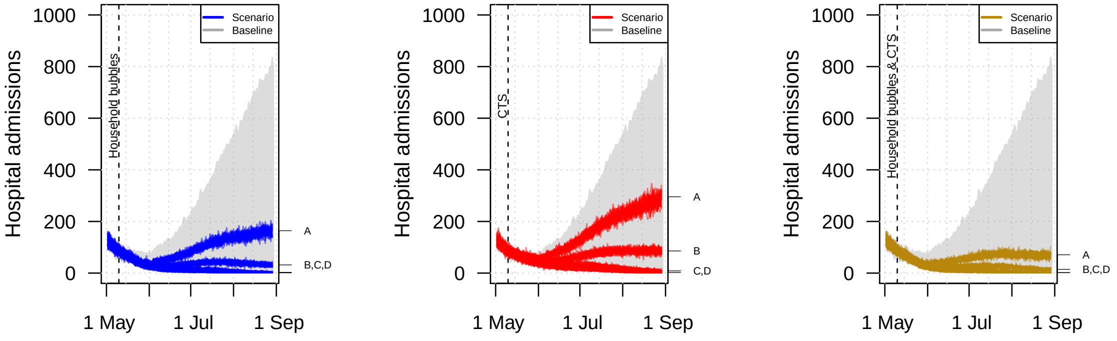

(b) Simulation results based on 40 stochastic realisations

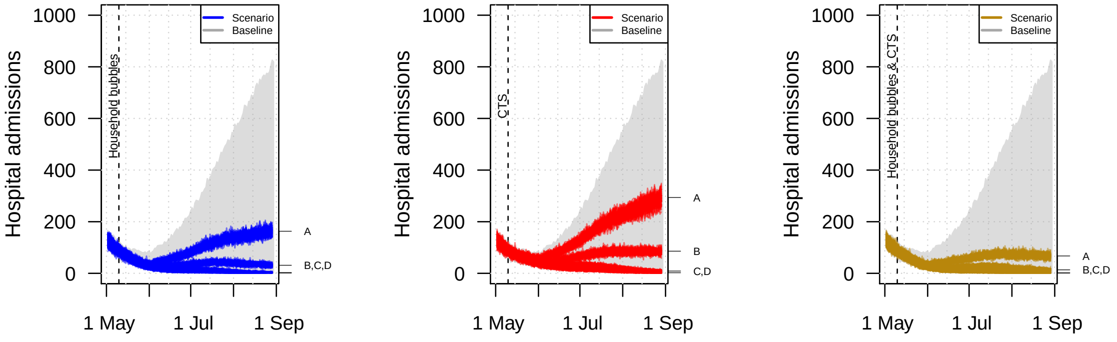

(c) Simulation results based on 80 stochastic realisations

Supplementary Figure 17: Hospital admissions over time when community mixing occurs in household bubbles (left), a contact tracing strategy (CTS) is in place (center), or both (right) based on 20, 40 and 80 stochastic realisations per social contact assumption. All scenarios are based on the same natural disease history and quantitative mixing assumptions but differ from the baseline in terms of the network structure and application of contact tracing from the given point in time. The mixing assumptions A,B,C,D are explained in the caption of Supplementary Figure 16..

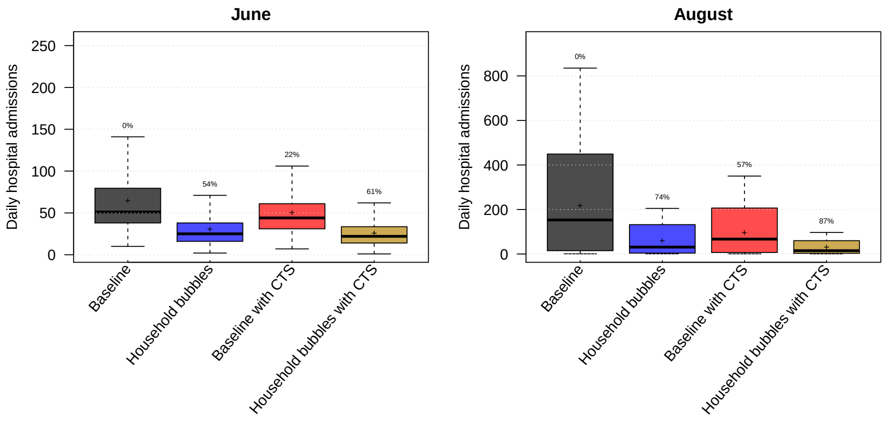

(a) Simulation results based on 20 stochastic realisations

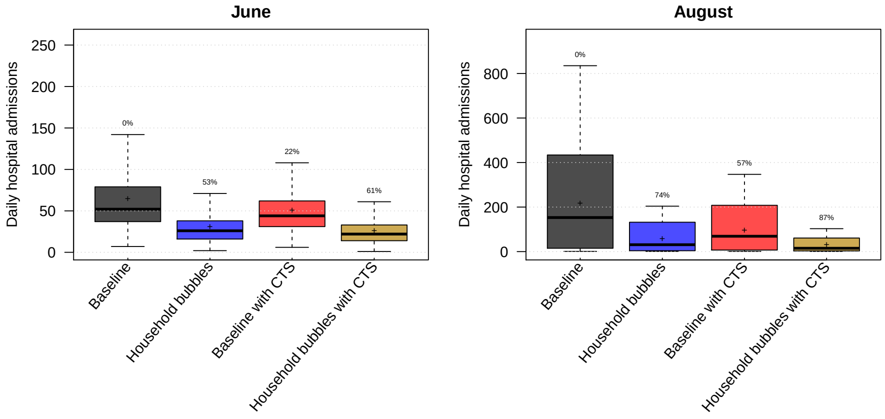

(b) Simulation results based on 40 stochastic realisations

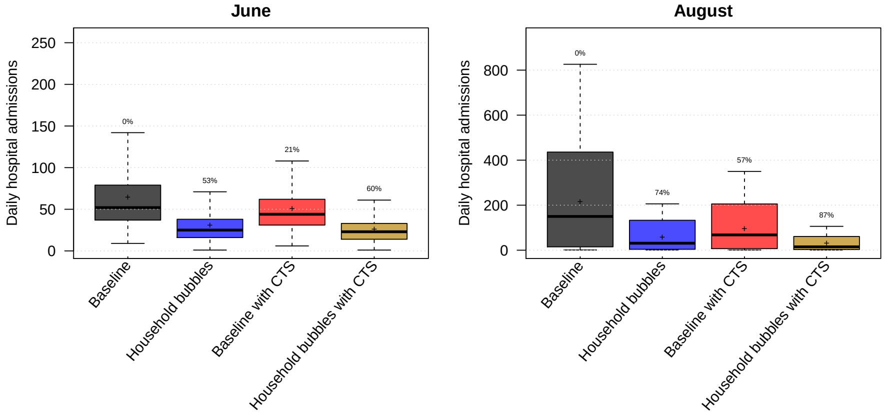

(c) Simulation results based on 80 stochastic realisations

Supplementary Figure 18: Distribution of the daily hospital admissions by June and August per scenario based on 20, 40 and 80 stochastic realisations per social contact assumption. The results are presented as the median (line), quartiles (box), 2.5 and 97.5 percentiles (whiskers) and average (cross) of 80, 160 and 240 model realisations (i.e., 20, 40 or 80 stochastic runs for each of the 4 social contact behaviour assumptions). The percentage on top of the whiskers indicates relative reduction of the scenario average with respect to the baseline. CTS: contact tracing strategy.

S11 Ensemble analyses

The scenario analyses presented in the main text are based on single parameter estimations from the iterative parameter estimation procedure (Section S7). Given the correlated nature of different model parameters, different combinations can give a similar fit for the first wave, but lead to different outcomes in terms of the scenario analyses. To endorse our results, we ran the main scenarios with all parameter sets from the model ensemble from the 4th iteration with and without age-specific susceptibility. The resulting hospital admissions over time in Supplementary Figure 19 and 20 include more variation in the hospital admissions over time. The average reductions in hospital admissions up to August, as presented in Supplementary Figure 21, are similar to the results presented in our main analysis (Supplementary Figure ??). We conclude that our results in terms of aggregated statistics based on our most optimal parameter set remain valid when we include parameter uncertainty.

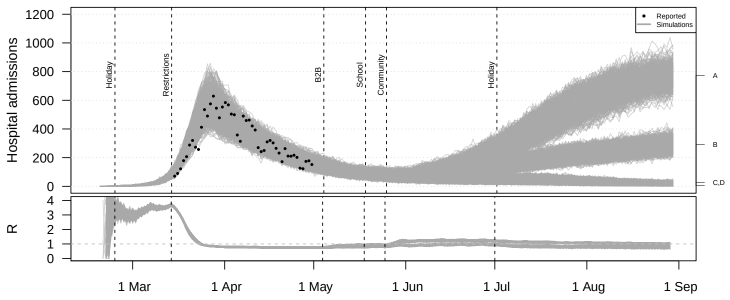

(a) Baseline scenario without age-specific susceptibility.

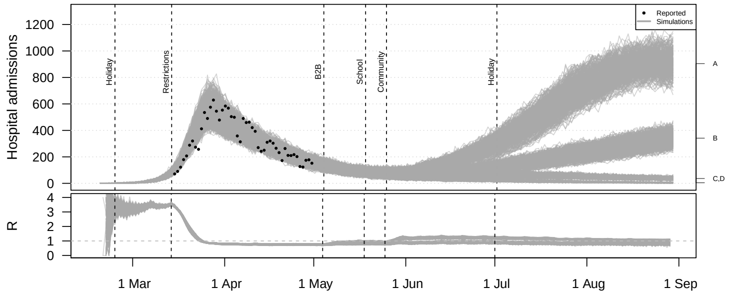

(b) Baseline [child] scenario with age-specific susceptibility.

Supplementary Figure 19: Hospital admissions over time for the baseline scenario based on the model parameter ensemble. All simulations include social restrictions from March 14th and the partial school reopening in May. For the B2B, the social mixing after the lockdown is assumed to double from the indicated point in time (indicated with A and C) or to remain constant (B,D). Social mixing in the community is assumed to double (A,B) or to remain constant (C,D). The dots present the reported hospital admissions for Belgium.

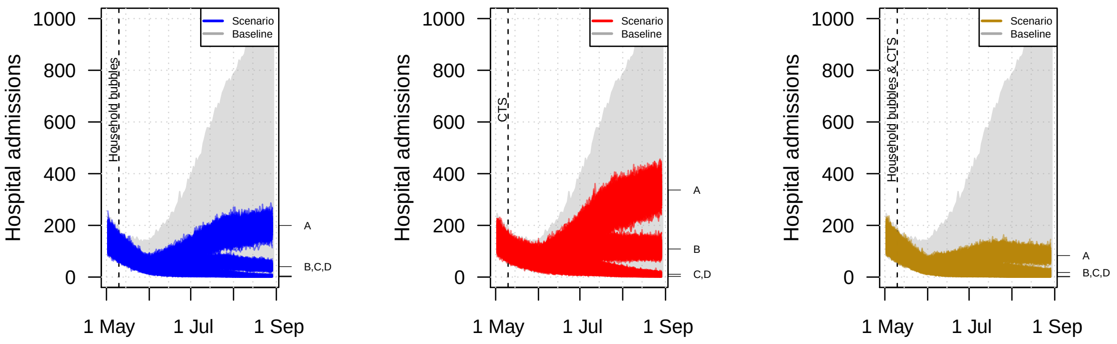

(a) Simulation results without age-specific susceptibility.

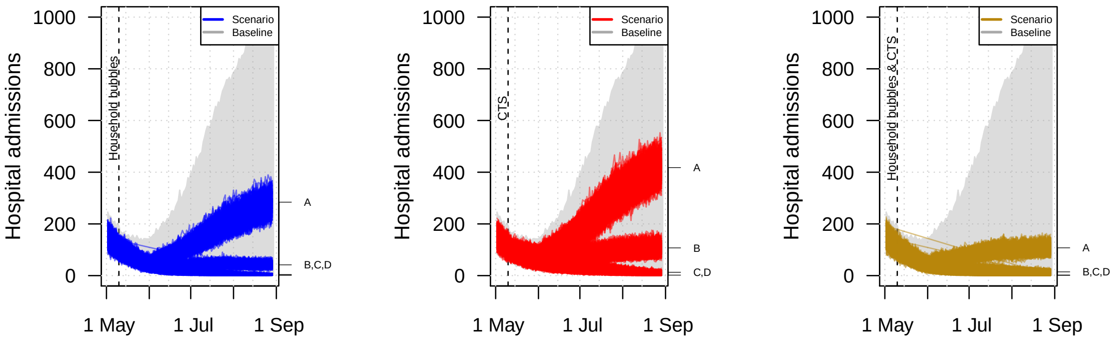

(b) Simulation results with age-specific susceptibility

Supplementary Figure 20: Hospital admissions over time when community mixing occurs in household bubbles (left), a contact tracing strategy (CTS) is in place (center), or both (right) based on the model parameter ensemble. All scenarios are based on the same natural disease history and quantitative mixing assumptions but differ from the baseline in terms of the network structure and application of contact tracing from the given point in time. The mixing assumptions A,B,C,D are explained in the caption of Supplementary Figure 19.

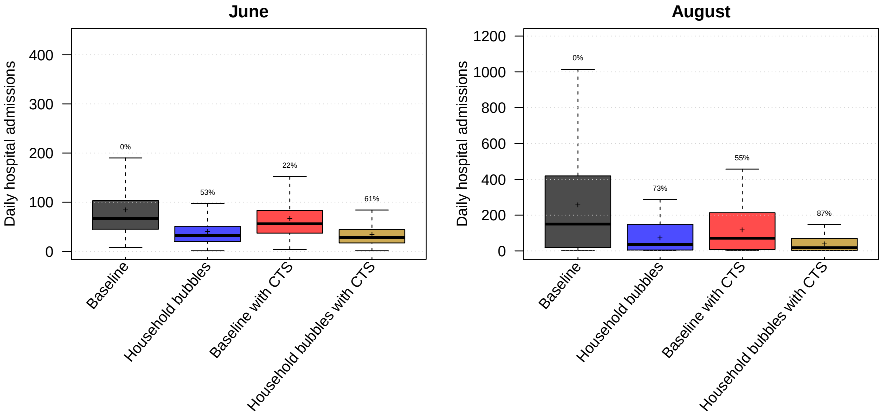

(a) Simulation results without age-specific susceptibility.

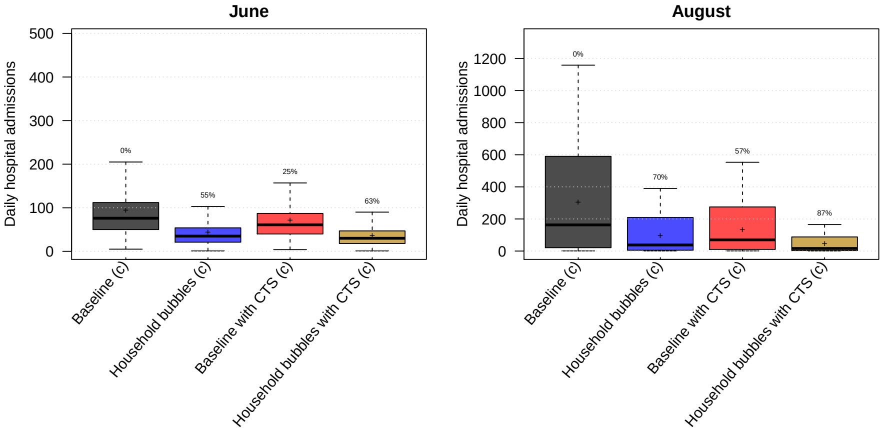

(b) Simulation results with age-specific susceptibility

Supplementary Figure 21: Distribution of the daily hospital admissions by June and August per scenario based on the model parameter ensemble. The results are presented as the median (line), quartiles (box), 2.5 and 97.5 percentiles (whiskers) and average (cross) of 40 model realisations (i.e., 10 stochastic runs for each of the 4 social contact behaviour assumptions). The percentage on top of the whiskers indicates relative reduction of the scenario average with respect to the baseline. CTS: contact tracing strategy.

S12 Platform and technical details

STRIDE is open-source software (`https://github.com/lwillem/stride_covid19_v1`) [23] and implemented in C++. The software can be compiled on Linux and Mac OSX platforms with a recent version of a C++ compiler. To build and install STRIDE, the following tools need to be available on the system: GNU g++ or LLVM clang++ compiler, make, CMake and Boost. To generate documentation, Doxygen and LaTeX are required. The build system for STRIDE uses the CMake tool to compile and install the software almost platform independent. More info is provided in the user manual on Github.

Our model is optimized with a switch to evaluate only the social contacts of the infectious individuals instead of matching every individual with all others within one location. More details on our model optimisations are provided in [1].

The modelling project contains regression tests in the Google Test suite embedded in a Travis continuous integration environment. The test environment has been created and maintained during model development for influenza and measles, and additional unit tests were added during this analysis. We implemented a baseline COVID-19 test and also benchmark physical distancing, household bubbles and contact tracing scenarios.

We implemented an "rSTRIDE" framework in R to handle the design of experiments, to run all parameter sets and analyse the output. Different serial STRIDE simulations are run in parallel using the "doParallel" package and the aggregation of summary statistics, prevalence, incidence and social contacts is automated. The synthetic population of 11 million individuals is computed once using R and loaded onto the C++ simulator for every new simulation. The synthetic population data we used for Belgium are available on ZENODO `https://doi.org/10.5281/zenodo.4485995`.

All results presented in this manuscript are generated on the VSC-cluster "Vaughan", a NEC system consisting of 104 nodes with two 32-core AMD Epyc 7452 Rome generation CPUs connected through a HDR100 InfiniBand network. All nodes have 256 GB RAM. One single run from the baseline scenario (196 days) required ±20 minutes.

S13 References (Supplementary Information)

[1] Willem, L., Stijven, S., Tijskens, E., Beutels, P., Hens, N., Broeckhove, J.: Optimizing agent-based transmission models for infectious diseases. BMC Bioinformatics **16**, 183 (2015)

[2] Kuylen, E., Stijven, S., Broeckhove, J., Willem, L.: Social contact patterns in an individual-based simulator for the transmission of infectious diseases (Stride). In: ICCS, pp. 2438–2442 (2017)

[3] Kuylen, E., Willem, L., Broeckhove, J., Beutels, P., Hens, N.: Clustering of susceptible individuals within households can drive measles outbreaks: an individual-based model exploration. Sci Rep **10**(19645) (2020)

[4] EUROSTAT: Your Key to European Statistics. `https://ec.europa.eu/eurostat/data/`

[5] Kind en Gezin. Vlaamse Overheid: Opvangadressen [status 2019-09-24]. https://www.kindengezin.be/toepassingen/zoekopvang.jsp (2019)

[6] Onderwijs Vlaanderen: Publicaties omkadering lestijden gewoon basisonderwijs 2012-2013. https://www.agodi.be/nieuwe-omkadering-basisonderwijs (2013)

[7] Willem, L., Van Kerckhove, K., Chao, D.L., Hens, N., Beutels, P.: A nice day for an infection? Weather conditions and social contact patterns relevant to influenza transmission. PLoS One **7**(11) (2012)

[8] Kifle, Y.W., Goeyvaerts, N., Van Kerckhove, K., Willem, L., Kucharski, A., Faes, C., Leirs, H., Hens, N., Beutels, P.: Animal ownership and touching enrich the context of social contacts relevant to the spread of human infectious diseases. PloS One **10**(7), 0133461 (2015)

[9] Van Hoang, T., Coletti, P., Kiffle, Y.W., Van Kerckhove, K., Vercruysse, S., Willem, L., Beutels, P., Hens, N.: Close contact infection dynamics over time: insights from a second large-scale social contact survey in Flanders, Belgium, in 2010-2011. medRxiv (2020)

[10] Goeyvaerts, N., Santermans, E., Potter, G., Torneri, A., Van Kerckhove, K., Willem, L., Aerts, M., Beutels, P., Hens, N.: Household members do not contact each other at random: implications for infectious disease modelling. Proc R Soc B **285**(1893), 20182201 (2018)

[11] Funk, S.: socialmixr: Social Mixing Matrices for Infectious Disease Modelling. The Comprehensive R Archive Network (2020)

[12] Willem, L., Hoang, V.T., Funk, S., Coletti, P., Beutels, P., Hens, N.: SOCRATES: an online tool leveraging a social contact data sharing initiative to assess mitigation strategies for COVID-19. BMC Res Notes **13**(1), 293 (2020)

[13] Li, Q., Guan, X., Wu, P., Wang, X., Zhou, L., Tong, Y., Ren, R., Leung, K.S.M., Lau, E.H.Y., *et al.*: Early transmission dynamics in Wuhan, China, of novel coronavirus–infected pneumonia. N Engl J Med **382**(13), 1199–1207 (2020)

[14] Wu, J.T., Leung, K., Bushman, M., Kishore, N., Niehus, R., de Salazar, P.M., Cowling, B.J., Lipsitch, M., Leung, G.M.: Estimating clinical severity of COVID-19 from the transmission dynamics in Wuhan, China. Nat Med, 1–5 (2020)

[15] He, X., Lau, E.H., Wu, P., Deng, X., Wang, J., Hao, X., Lau, Y.C., Wong, J.Y., Guan, Y., Tan, X., *et al.*: Temporal dynamics in viral shedding and transmissibility of COVID-19. Nat Med **26**(5), 672–675 (2020)

[16] Lourenco, J., Paton, R., Ghafari, M., Kraemer, M., Thompson, C., Simmonds, P., Klenerman, P., Gupta, S.: Fundamental principles of epidemic spread highlight the immediate need for large-scale serological surveys to assess the stage of the SARS-CoV-2 epidemic. MedRxiv (2020)

[17] Li, R., Pei, S., Chen, B., Song, Y., Zhang, T., Yang, W., Shaman, J.: Substantial undocumented infection facilitates the rapid dissemination of novel coronavirus (SARS-CoV-2). Science **368**(6490), 489–493 (2020)

[18] Faes, C., Abrams, S., Van Beckhoven, D., Meyfroidt, G., Vlieghe, E., Hens, N.: Time between symptom onset, hospitalisation and recovery or death: Statistical analysis of Belgian COVID-19 patients. Int J Environ Res Public Health **17**(20), 7560 (2020)

[19] Sciensano, Belgium: COVID-19 - Epidemiologische situatie. https://epistat.wiv-isp.be/covid/ (2020)

[20] Pellis, L., Scarabel, F., Stage, H.B., Overton, C.E., Chappell, L.H., Lythgoe, K.A., Fearon, E., Bennett, E., Curran-Sebastian, J., Das, R., et al.: Challenges in control of Covid-19: short doubling time and long delay to effect of interventions. arXiv:2004.00117 (2020)

[21] Herzog, S., De Bie, J., Abrams, S., Wouters, I., Ekinci, E., Patteet, L., Coppens, A., De Spiegeleer, S., Beutels, P., Van Damme, P., Hens, N., Theeten, H.: Seroprevalence of IgG antibodies against SARS coronavirus 2 in Belgium: a serial prospective cross-sectional nationwide study of residual samples. medRxiv (2020)

[22] Hilbe, J.: Modeling Count Data., (2014)

[23] Willem, L., Kuylen, E., Libin, P.J.K., Hens, N.: STRIDE (v1.1.0 Household Bubbles). `https://doi.org/10.5281/zenodo.4440258`
