# Supplementary material for: The impact of contact tracing and household bubbles on deconfinement strategies for COVID-19
Source: Nat Commun. 2021 Mar 9;12:1524. doi: 10.1038/s41467-021-21747-7 (PMC7943552; doi:10.1038/s41467-021-21747-7)
Supplement: Supplementary file 3 — Reporting Summary [file 41467_2021_21747_MOESM3_ESM.pdf]

nature research

Corresponding author(s): Lander Willem

Last updated by author(s): Jan 28, 2021

Reporting Summary

Nature Research wishes to improve the reproducibility of the work that we publish. This form provides structure for consistency and transparency in reporting. For further information on Nature Research policies, see our Editorial Policies and the Editorial Policy Checklist.

Statistics

For all statistical analyses, confirm that the following items are present in the figure legend, table legend, main text, or Methods section.

n/a	Confirmed	
☐	☒	The exact sample size (*n*) for each experimental group/condition, given as a discrete number and unit of measurement
☐	☒	A statement on whether measurements were taken from distinct samples or whether the same sample was measured repeatedly
☒	☐	The statistical test(s) used AND whether they are one- or two-sided<br>*Only common tests should be described solely by name; describe more complex techniques in the Methods section.*
☒	☐	A description of all covariates tested
☒	☐	A description of any assumptions or corrections, such as tests of normality and adjustment for multiple comparisons
☐	☒	A full description of the statistical parameters including central tendency (e.g. means) or other basic estimates (e.g. regression coefficient) AND variation (e.g. standard deviation) or associated estimates of uncertainty (e.g. confidence intervals)
☒	☐	For null hypothesis testing, the test statistic (e.g. *F*, *t*, *r*) with confidence intervals, effect sizes, degrees of freedom and *P* value noted<br>*Give P values as exact values whenever suitable.*
☒	☐	For Bayesian analysis, information on the choice of priors and Markov chain Monte Carlo settings
☒	☐	For hierarchical and complex designs, identification of the appropriate level for tests and full reporting of outcomes
☒	☐	Estimates of effect sizes (e.g. Cohen's *d*, Pearson's *r*), indicating how they were calculated

Our web collection on statistics for biologists contains articles on many of the points above.

Software and code

Policy information about availability of computer code

Data collection	Data regarding the daily number of COVID-19 cases and hospital admissions were made available by Belgian Institute of Public Health (Sciensano) by means of CSV spreadsheets. Thus, no specific software was used to collect this data.
Data analysis	No specific software was used for data analysis. However, we provide all code and model configuration scripts in a public archive of our open-source GitHub repository (https://doi.org/10.5281/zenodo.4440258). The synthetic population data we used for Belgium are also available on ZENODO (https://doi.org/10.5281/zenodo.4485995).

For manuscripts utilizing custom algorithms or software that are central to the research but not yet described in published literature, software must be made available to editors and reviewers. We strongly encourage code deposition in a community repository (e.g. GitHub). See the Nature Research guidelines for submitting code & software for further information.

Data

Policy information about availability of data

All manuscripts must include a data availability statement. This statement should provide the following information, where applicable:

- Accession codes, unique identifiers, or web links for publicly available datasets
- A list of figures that have associated raw data
- A description of any restrictions on data availability

The series of COVID-19 cases and hospital admissions are publicly available from the Belgian Institute of Public Health (Sciensano) at https://epistat.wiv-isp.be/covid/covid-19.html and are also available as CSV files. All other data to inform the model are available at doi:10.5281/zenodo.4440258 and doi:10.5281/zenodo.4485995.

Field-specific reporting

Please select the one below that is the best fit for your research. If you are not sure, read the appropriate sections before making your selection.

- [x] Life sciences
- [ ] Behavioural & social sciences
- [ ] Ecological, evolutionary & environmental sciences

For a reference copy of the document with all sections, see nature.com/documents/nr-reporting-summary-flat.pdf

Life sciences study design

All studies must disclose on these points even when the disclosure is negative.

Sample size	Since this is a mathematical modeling study, sample size information is not presented.
Data exclusions	No data were excluded from the time series of cases and hospital admissions that were used to fit the model.
Replication	Replication of our findings were assured by the use of multiple stochastic realisations of the simulation model to define uncertainty intervals of the predictions. To validate our choice for presenting results based on 10 stochastic realisations, we also ran our main scenarios (baseline, household bubbles and contact-tracing) with 20, 40 and 80 stochastic iterations. We observed stochastic differences in the hospital admissions over time, but the results in terms of the average number of hospital admissions did not alter. We also ran simulations using an ensemble of estimated model parameters and observed again stochastic differences in the hospital admissions over time, but stable results in terms of the average number of hospital admissions.
Randomization	Since this is a mathematical modeling study, randomization procedures were not required.
Blinding	Since this is a mathematical modeling study, blinding procedures were not required.

Reporting for specific materials, systems and methods

We require information from authors about some types of materials, experimental systems and methods used in many studies. Here, indicate whether each material, system or method listed is relevant to your study. If you are not sure if a list item applies to your research, read the appropriate section before selecting a response.

Materials & experimental systems

n/a	Involved in the study	
☒	☐	Antibodies
☒	☐	Eukaryotic cell lines
☒	☐	Palaeontology and archaeology
☒	☐	Animals and other organisms
☒	☐	Human research participants
☒	☐	Clinical data
☒	☐	Dual use research of concern

Methods

n/a	Involved in the study	
☒	☐	ChIP-seq
☒	☐	Flow cytometry
☒	☐	MRI-based neuroimaging
